# Double Maximum Ratios of Viruses to Bacteria in the Water Column: Implications for Different Regulating Mechanisms

**DOI:** 10.3389/fmicb.2019.01593

**Published:** 2019-07-16

**Authors:** Lei He, Kedong Yin, Xiangcheng Yuan

**Affiliations:** ^1^School of Marine Sciences, Sun Yat-sen University, Guangzhou, China; ^2^Guangdong Provincial Key Laboratory of Marine Resources and Coastal Engineering, Guangzhou, China; ^3^Southern Marine Science and Engineering Guangdong Laboratory (Zhuhai), Zhuhai, China; ^4^Key Laboratory of Marine Bio-Resources Sustainable Utilization, South China Sea Institute of Oceanology, Chinese Academy of Sciences, Guangzhou, China

**Keywords:** vertical distribution, marine virus, marine bacterium, nutrients, oxygen minimum zone, South China Sea

## Abstract

The viruses play an important role in limiting bacterial abundance in oceans and, hence, in regulating bacterial biogeochemical functions. A cruise was conducted in September 2005 along a transect in the deep South China Sea (SCS). The results showed the double maxima in the ratio of viral to bacterial abundance (VBR) in the water column: a deep maximum at 800–1000 m coinciding with the oxygen minimum zone (OMZ) and a subsurface maximum at 50–100 m near the subsurface chlorophyll maximum (SCM) layer. At the deep maximum of VBR, both viral and bacterial abundances were lower than those in the upper layer, but the former was reduced less than the latter. In contrast, at the subsurface maximum of VBR, both viral and bacterial abundances increased to the maximum, with viral abundance increasing more than bacterial abundance. The results suggest that two VBR maxima were formed due to different mechanisms. In the SCM, the VBR maximum is due to an abundant supply of organic matter, which increases bacterial growth, and stimulates viral abundance faster. In contrast, in the OMZ, organic matter is consumed and limits bacterial growth, but viruses are less limited by organic matter and continue to infect bacteria, leading to the maximum VBR. The OMZ in the deep-water column of oceans is over hundreds of years old and receives a constant supply of organic matter from the water above. However, the oxygen level cannot be depleted to anoxia. Bacterial respiration is largely responsible for oxygen consumption in the OMZ; and hence, any process that limits bacterial abundance and respiration contributes to the variation in the OMZ. Viral control of bacterial abundance can be a potential mechanism responsible for slowing down oxygen consumption to anoxia in the OMZ. Our finding provides preliminary evidence that viruses are an important player in controlling bacterial abundance when bacterial growth is limited by organic matter, and thus, regulates the decomposition of organic matter, oxygen consumption and nutrient re-mineralization in deep oceans.

## Introduction

The viral shunt plays an important role in biogeochemical processes in oceans (Suttle, 2005). Viruses are present at concentrations in the levels of 10^8^ ml^–1^ in coastal waters, 10^7^ ml^–1^ in offshore waters and 10^6^ ml^–1^ in oceanic waters, one order of magnitude higher than the bacterial abundance for coastal, offshore and oceanic waters, respectively. Most observations of viruses are focused on surface waters. Measurements of oceanic viral-particle direct counts in the deep oceans (>1000 m) are relatively scarce, and they have been conducted only in regions of the Southeastern Gulf of Mexico (Boehme et al., 1993), the Mediterranean Sea (Magagnini et al., 2007; Winter et al., 2009; Fonda Umani et al., 2010; Magiopoulos and Pitta, 2012), the North Pacific (Hara et al., 1996), the Atlantic (Parada et al., 2007; De Corte et al., 2010; De Corte et al., 2012; De Corte et al., 2016), the Southern Oceans (Yang et al., 2014), and the tropical and subtropical waters of the global ocean (Lara et al., 2017). The subsurface maximum of viral abundance has often been observed, for example, at 50 m in the southeastern Gulf of Mexico (Boehme et al., 1993) and in the North Pacific (Hara et al., 1996), at 75–100 m at all stations of the Ionian, Libyan and South Aegean Seas (Magiopoulos and Pitta, 2012) and 50–100 m in the Mediterranean Sea (Magagnini et al., 2007). In the North Aegean Sea, the maximum viral abundance occurred at 2 to 50 m (Magiopoulos and Pitta, 2012). The ratio of viral to bacterial abundance (VBR) is often used as an indicator for the relationship between bacteria and viruses. The ratio of viral abundance to prokaryotic abundance is the result of a comprehensive balance of factors, such as the viral production, the transport of viruses through sinking particles, decay rates and life strategies (Hara et al., 1996; Weinbauer et al., 2003; Wigington et al., 2016).

Viruses affect bacterial ecological functions such as decomposition and respiration of organic matter, and thus, play an important role in biogeochemical processes in the deep ocean. A previous study (Liu et al., 2015) has shown that viruses reduce bacterial abundance and bacterial respiration in laboratory cultures compared with virus-free bacterial cultures. However, such viral effects depend on the nutrient conditions. Viruses exert more control on bacterial abundance and activities in eutrophic seawater, but viruses sustain the bacterial population by viral lysates of bacteria under nutrient-limited seawater. This clearly suggests that viruses play a more important role where the organic supply is limited. Dissolved oxygen (DO) is a key indicator for biological activity and biogeochemical processes in deep oceans. Because of the thermohaline circulation in the ocean and biological consumption of oxygen, an oxygen minimum zone (OMZ) exists in the middle water column of the deep oceans. The OMZ feature occurs in most world oceans. The formation of an OMZ is thought to result from the balance of organic-matter-sustained oxygen consumption and the thermohaline circulation (Paulmier and Ruiz-Pino, 2009). Given the long residence time of the OMZ water mass, DO should be depleted; however, DO has rarely been depleted in the OMZ. We hypothesize that viruses play a regulating role in controlling bacterial abundance and the bacterial growth is limited by the organic matter in the water column. To test this hypothesis, a cruise was conducted in the northern South China Sea (SCS).

The SCS is the largest inland sea in the tropical region, extending from the equator to 22°N and from 99°E to 121°E, with a surface area of approximately 3.5 × 10^6^ km^2^. The maximum water depth is approximately 5000 m (Shaw and Chao, 1994), with the average depth being approximately 1200 m. The SCS exchanges waters with the Pacific and Indian Oceans through several passages, most of which are very shallow (less than 100 m). Only the Luzon Strait has a depth of approximately 2500 m and allows more exchange of deeper waters in the Philippine Sea between the western Pacific and SCS (Qu et al., 2006; Wang et al., 2018). The SCS is oligotrophic with a high sea-surface temperature, low nutrients, low chlorophyll *a*, and low primary productivity (Liu et al., 2002; Wu et al., 2003; Ning et al., 2005), but relatively high viral and bacterial abundance (He et al., 2009). However, there is little information on the occurrence and characteristics of viruses and bacteria in the deep oceanic water column in the SCS. The major objectives of this study were to test the hypothesis by investigating the vertical distribution of viruses and bacteria and to examine the role of viruses in regulating bacterial abundance and biogeochemical processes in the deep ocean water column in the SCS.

## Materials and Methods

### Sampling Stations and Sampling

**FIGURE 1:**
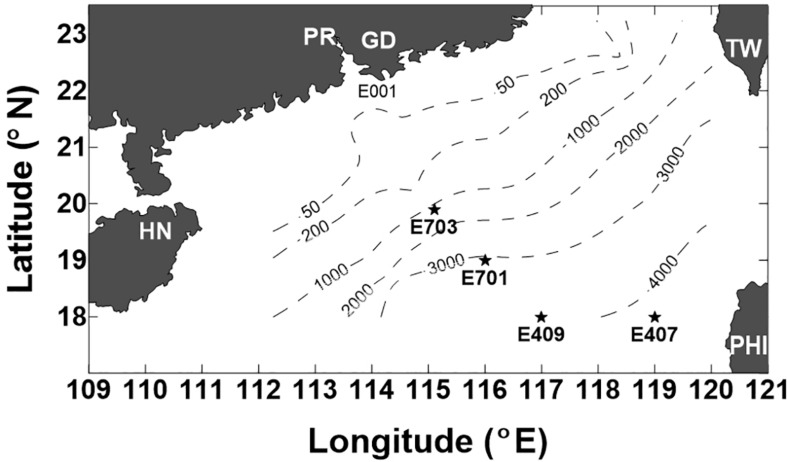
Location of the stations sampled in the northern South China Sea (SCS) during the cruise of 5–23 September 2005 (PR-Pearl River, GD-Guangdong, HN-Hainan, TW-Taiwan, and PHI-Philippines). Dashed lines indicate the depth contours of 50 m, 200 m, 1000 m, 2000 m, 3000 m, and 4000 m.

A cruise on board the R/V *Experiment No. 3* was conducted in the northern SCS during September 5–23, 2005. Four deep stations were visited (Figure 1): E703 (water depth 1182 m, 19°54′N, and 115°06′E), E701 (water depth 3099 m, 18°59′N, and 116°00′E), E409 (water depth 3945 m, 17°59′N, and 116°59′E), and E407 (water depth 4150 m, 17°59′N, and 119°00′E). The sampling depths covered the entire water column from the surface to 4000 m. A CTD rosette with 12 Niskin bottles was used to read vertical profiles of the salinity and temperature. Water samples were collected at twelve discrete sampling depths throughout the water column: three in the epipelagic zone (3, 50, and 100 m), four in the mesopelagic zone (200, 300, 500, and 800 m) and five in the bathypelagic zone (1000, 1500, 2000, 3000, and 4000 m). A YSI^@^ 6600 probe was used and slowly lowed into the water column, taking readings of chlorophyll fluorescence, down to 100 m. Samples for DO were collected following the water overflow procedure, and DO was determined by the Winkler titration method as outlined by Parsons et al. (1984).

### Counting of Viruses and Bacteria

Samples for bacterial and viral abundance were transferred to 2 ml centrifuge tube, fixed immediately with glutaraldehyde (final concentration, 0.5%) and stored in darkness for 15 min. Fixed for 15 min, 0.8 ml samples were filtered onto 0.02 μm Anodisc filters (Whatman, Maidstone, United Kingdom). The vacuum pressure was about 20 kPa. The filters were placed on a drop of SYBR-Green-I solution (final concentration, 0.25%) and dyed in darkness for 15 min, according to Noble and Fuhrman (1998). The slides with the fixed filters were completed in about 30 min and stored at −20°C. The counting of bacteria and viruses was made within 4 weeks. With the immersion oil (type A, Nikon), Virus and bacterial particles were counted at 1000 times magnification under 100 W mercury lamp for the epifluorescence illumination using an OLYMPUS BX41 microscope in the laboratory. At least 200 bacteria and viruses in each field were counted in at least 10 fields (Noble and Fuhrman, 1998).

### Nutrients

Nitrite samples were filtered through glass fiber filters (GF/F) and frozen immediately (−20°C) until analysis. All plastic-wares were pre-cleaned with 10% HCl. The nitrite was analyzed colorimetrically with a SKALAR (San Plus) Autoanalyzer using JGOFS Protocols (Knap et al., 1996). Dissolved organic carbon (DOC) samples were filtered through pre-combusted 0.7 μm GF/F filters. The DOC samples were acidified with 50 μl 50% H_3_PO_4_ to pH < 2 to remove the inorganic carbon, and the acidified samples were purged with ultra-high purity nitrogen for about 10 min before analysis to drive off the inorganic carbon (Knap et al., 1996).

### Statistical Analysis

Spearman correlation coefficient was used to test the relationships between variables. By using an analysis of variance and a Tukey post-test, the significant differences for all parameters between depth zones were studied. All statistical analyses and the principal components analysis (PCA) were performed using SPSS statistics 19.0 software (SPSS Inc.).

## Results

### Vertical Profiles of Salinity and Temperature

**FIGURE 2:**
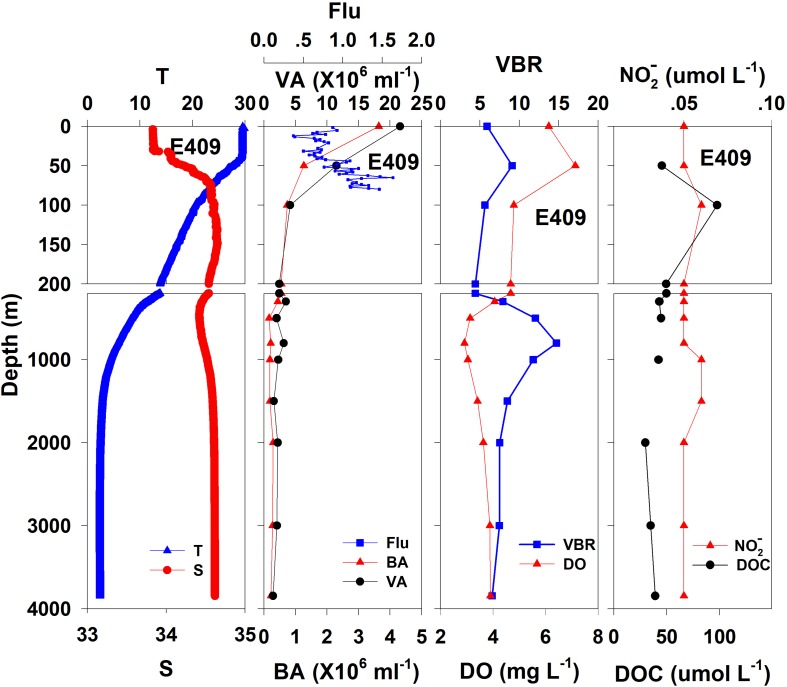
The vertical distributions of salinity (S, psu), temperature (T, °C), chlorophyll fluorescence, bacterial abundance (BA, 10^6^ ml^–1^), viral abundance (VA, 10^6^ ml^–1^), VBR, dissolved oxygen (DO, mg L^–1^), DOC (μmol L^–1^), and NO${}_{2}^{-}$ (μmol L^–1^) at E409.

**FIGURE 3:**
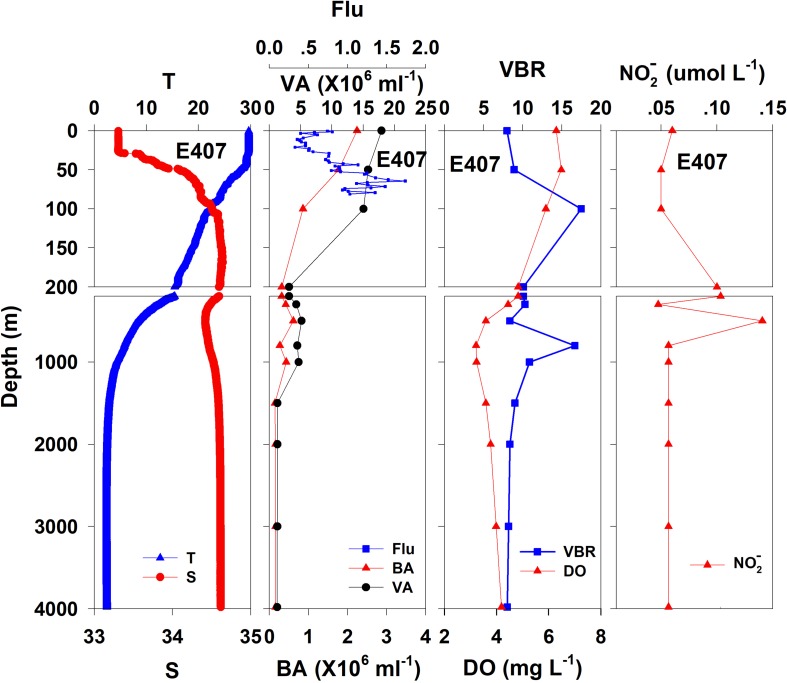
The vertical distributions of salinity (S, psu), temperature (T, °C), chlorophyll fluorescence, bacterial abundance (BA, 10^6^ ml^–1^), viral abundance (VA, 10^6^ ml^–1^), VBR, dissolved oxygen (DO, mg L^–1^), and NO${}_{2}^{-}$ (μmol L^–1^) at E407.

**FIGURE 4:**
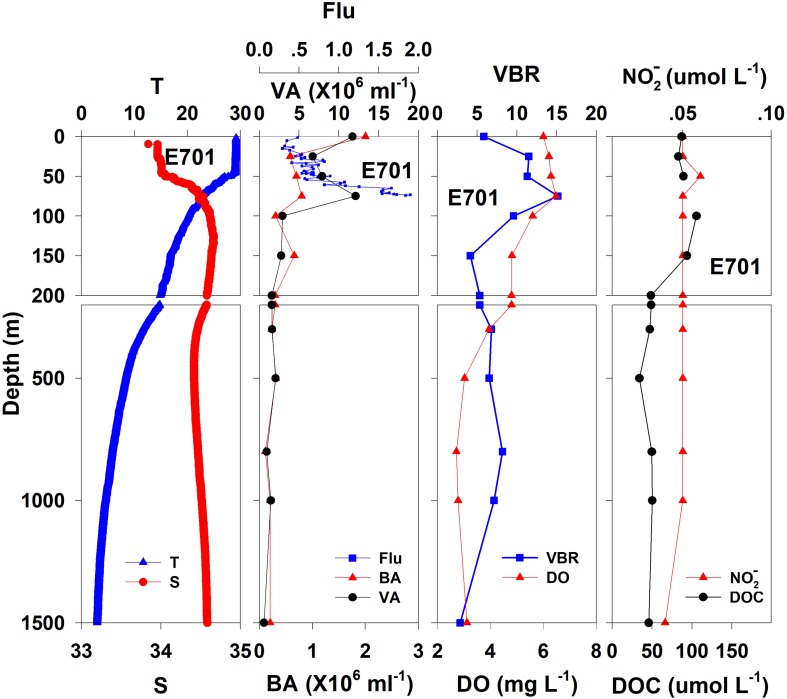
The vertical distributions of salinity (S, psu), temperature (T, °C), chlorophyll fluorescence, bacterial abundance (BA, 10^6^ ml^–1^), viral abundance (VA, 10^6^ ml^–1^), VBR, dissolved oxygen (DO, mg L^–1^), DOC (μmol L^–1^), and NO${}_{2}^{-}$ (μmol L^–1^) at E701.

**FIGURE 5:**
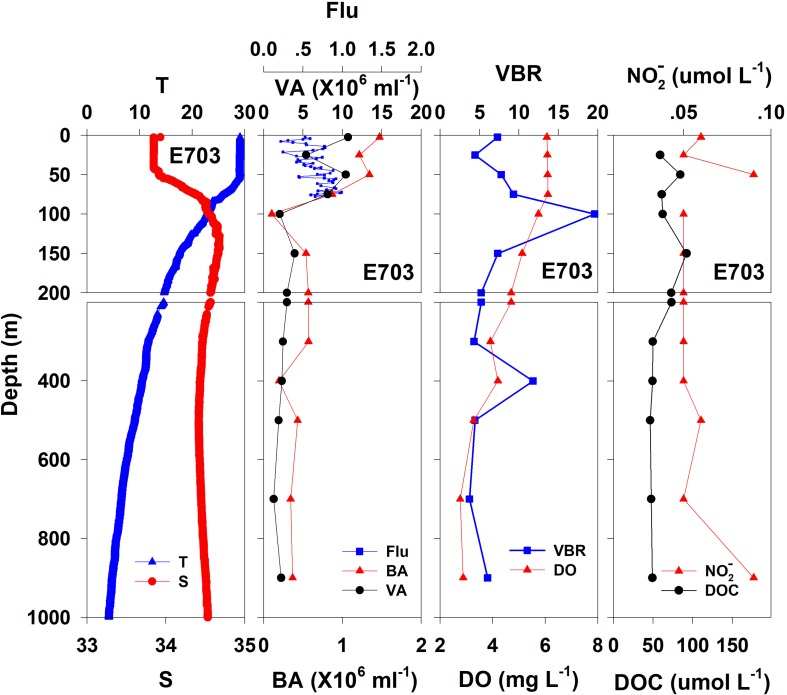
The vertical distributions of salinity (S, psu), temperature (T, °C), chlorophyll fluorescence, bacterial abundance (BA, 10^6^ ml^–1^), viral abundance (VA, 10^6^ ml^–1^), VBR, dissolved oxygen (DO, mg L^–1^), DOC (μmol L^–1^), and NO${}_{2}^{-}$ (μmol L^–1^) at E703.

The salinity was 33.97 in the mixed layer of approximately 30 m at E409 (Figure 2), and the halocline depth was 70 m at E409. Below 70 m, the salinity increased gradually and reached a maximum (34.65) at approximately 150 m throughout the water column. There was a salinity minimum of 34.42 at 445 m from which the salinity increased to 34.60 at approximately 1500 m. The salinity was constant below 1500 m. The temperature was 29.50°C in the mixed layer of approximately 30 m at E409. The thermocline was thick; the temperature decreased with depth within the mesopelagic zone, reaching 4.42°C at 1000 m and 2.50°C at 2000 m, below which temperature was constant throughout the bathypelagic layer (2.40°C). The vertical distribution of salinity and temperature at the other deep stations E407 (Figure 3), E701 (Figure 4), and E703 (Figure 5) was similar to that at E409 except for variations in the depth of the mixed layer, the thickness of the halocline and the depth of the salinity minimum. The average values of salinity and temperature among stations are summarized for the epipelagic, mesopelagic and bathypelagic zones (Table 1).

**TABLE 1:** Average and standard deviation (SD) of environmental variables at E409, E407, E701, and E703.

**Layers**	**Depth(m)**	**Salinity (psu)**	**Temperature (°C)**	**VA (×10^6^ ml^–1^)**	**BA (×10^6^ ml^–1^)**
Epipelagic	0–200	34.13 ± 0.56	25.17 ± 4.28	9.07 ± 5.10	1.24 ± 0.77
Mesopelagic	200–1000	34.48 ± 0.06	10.17 ± 3.51	2.56 ± 1.15	0.35 ± 0.15
Bathypelagic	1000–4000	34.58 ± 0.04	3.03 ± 0.86	1.78 ± 1.03	0.22 ± 0.08

**Layers**	**Depth(m)**	**DO (mg L^–1^)**	**chlorophyll fluoresces (μg L^–1^)**	**DOC (μmol L^–1^)**	**VBR**

Epipelagic	0–200	5.89 ± 0.61	0.17 ± 0.10	74.12 ± 19.22	7.94 ± 3.75
Mesopelagic	200–1000	3.77 ± 0.76	–	48.45 ± 8.27	7.60 ± 3.41
Bathypelagic	1000–4000	3.54 ± 0.42	–	40.34 ± 7.47	7.58 ± 2.21

### Vertical Distribution of Viral Abundance, Bacterial Abundance, and VBR

#### Viral Abundance

In general, the viral abundance decreased from the surface to the deeper layer. At E409 (Figure 2), the viral abundance was 21.59 × 10^6^ ml^–1^ at the surface, decreased to 2.43 × 10^6^ ml^–1^ at 200 m and was lower below 200 m. However, there was a deep maximum (3.15 × 10^6^ ml^–1^) at 800 m. At E407, the deep maximum of viral abundance was more pronounced, running from 300 to 1000 m (Figure 3); however, the vertical distribution of viral abundance was similar to that at E409. At E701 (Figure 4) and E703 (Figure 5), there were one or two maximums above 100 m, and the viral abundance was low below 100 m.

#### Bacterial Abundance

At E409 (Figure 2), the bacterial abundance decreased sharply from 3.64 × 10^6^ ml^–1^ at the surface to 0.73 × 10^6^ ml^–1^ at 100 m, and the decrease was slower from 0.73 × 10^6^ ml^–1^ at 100 m to 0.17 × 10^6^ ml^–1^ at 500 m. The bacterial abundance was lower than 0.20 × 10^6^ ml^–1^ below 500 m. The vertical distribution of bacterial abundance at the other deep stations E703 (Figure 5), E701 (Figure 4), and E407 (Figure 3) was similar to that at E409.

#### VBR

The VBR value remained constant or decreased from the epipelagic to the mesopelagic and bathypelagic zones on average (Table 1). The distinct feature in the vertical distribution of bacterial and viral abundance were the occurrences of two maxima of VBR in the water column at the deep stations: the subsurface maximum between 50 and 100 m and the deep maximum at approximately 800 m. At E409 (Figure 2), VBR was 8.21 at the subsurface maximum and higher (12.33) at the deep maximum. The double maximum also occurred at E407 (Figure 3), E701 (Figure 4), and E703 (Figure 5).

### Chemical and Biological Parameters

The DO in the epipelagic zone was higher than that in the mesopelagic and bathypelagic zones (Table 1). At the deep station E409 (Figure 2), DO was 6.12 mg L^–1^ at the surface, increased to 7.13 mg L^–1^ at 50 m, decreased to 2.91 mg L^–1^ at 800 m, and increased again to 3.90 mg L^–1^ at 3800 m. A deep minimum for DO occurred at approximately 800 m. The vertical distribution of the other deep stations, E407 (Figure 3), E701 (Figure 4), and E703 (Figure 5), was similar to that at E409. In these deep stations, DO presented a maximum at the subsurface layer and a deep minimum at approximately 800 m. The chlorophyll fluorescence was 0.80 μg L^–1^ at the surface, increased to 1.68 μg L^–1^ at 65 m, and decreased to 1.20 μg L^–1^ at E409. For the other stations, the chlorophyll fluorescence values increased from the surface layer to the subsurface layer. The DOC in the epipelagic zone was higher than that in the mesopelagic and bathypelagic zones (Table 1). For the deep station E409 (Figure 2), DOC was 45.68 μmol L^–1^ at 50 m, increased to 98.27 μmol L^–1^, formed a maximum at 100 m, decreased to 49.75 μmol L^–1^ at 200 m, and remained at approximately 40 μmol L^–1^ below 200 m. The vertical distribution of DOC at E701 (Figure 4) was similar to that at E409. At E703 (Figure 5), there were two maximum DOC at 50 m and 150 m, and the DOC remained at 50.00 μmol L^–1^ below 300 m. Nitrite was lower at all layers. At the deep stations, E409 (Figure 2), E701 (Figure 4), and E703 (Figure 5), nitrite was below 0.10 μmol L^–1^ in the water column. At the deep station E407 (Figure 3), there were two maxima at 200 m (0.10 μmol L^–1^) and 500 m (0.14 μmol L^–1^), and the value remained low at other depths.

### Relationships Between Viruses and Other Variables

**FIGURE 6:**
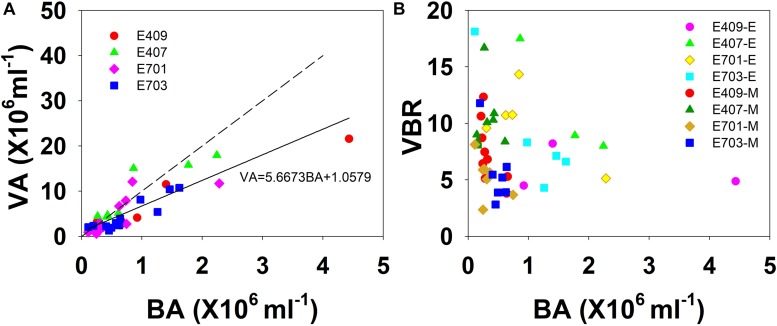
The relationship among VA, BA, and VBR. **(A)** Best fit for each study plot (solid line) and the 10:1 line (dashed line); **(B)** VBR is larger in the smaller bacterial abundance side in the mesopelagic and bathypelagic (E represents epipelagic zone, M represents mesopelagic, and bathypelagic zones).

The viral abundance was positively correlated with the bacterial abundance (Figure 6A, *r* = 0.874, *p* < 0.001, *n* = 48). The regression slope of VA against BA was 5.67, smaller than the 10:1 line (Figure 6A). Whether the correlations among VA, BA, and VBR are significant depends on the different layers. For the epipelagic zone, the correlation between VBR and BA (*r* = −0.454, *p* = 0.058, *n* = 18) was not significant at *p* < 0.05, but was stronger than that between VBR and VA (*r* = −0.039, *p* = 0.879, *n* = 18); meanwhile, in the mesopelagic-bathypelagic zone the correlation between VBR and BA was not significant (*r* = −0.299, *p* = 0.109, *n* = 30), but the correlation between VBR and VA (*r* = 0.504, *p* = 0.005, *n* = 30) was significant. This indicates that in the epipelagic zone, the variation in BA dominated the variation in VBR, whereas in the mesopelagic and bathypelagic zones, VA drove the variation in VBR (Figure 6B). However, no correlation was found between the viral abundance and chlorophyll *a* concentration (*r* = 0.201, *p* = 0.531, *n* = 12). A PCA was applied to the epipelagic and the mesopelagic-bathypelagic zones separately (Table 2). For the epipelagic zone, the first PC (PC1) had important contributions from chlorophyll, VBR and temperature, whereas PC2 was heavily loaded by the contributions from VA, BA, and DOC. PC3 had important contributions from salinity and nitrite, whereas PC4 was heavily loaded by the contributions from DO. For the mesopelagic and bathypelagic zones, PC1 was heavily loaded by the contributions from VA, VBR, nitrite and DOC; PC2 had significant contributions from BA, temperature and DO, whereas PC3 had significant contributions from salinity.

**TABLE 2:** Main results of the PCA application for all parameters in the epipelagic zone (a) and the mesopelagic and bathypelagic zones (b).

**Rotated Component Matrix^a^**				
**a**	**Component**			
	**1**	**2**	**3**	**4**
VA	–0.022	**0.948**	–0.204	–0.033
BA	–0.455	**0.843**	–0.163	–0.087
Chla	**0.931**	0.189	–0.131	–0.211
VBR	**0.918**	–0.259	0.020	0.115
S	0.573	–0.014	**0.643**	0.161
T	−**0.816**	0.036	–0.413	–0.192
DO	0.021	0.088	–0.046	**0.971**
NO2	–0.056	–0.268	**0.891**	–0.144
DOC	–0.237	−**0.820**	0.014	–0.356
Eigenvalue	3.48	2.484	1.142	1.013
Variance (%)	38.668	27.6	12.689	11.251
Cumulative variance(%)	38.668	66.268	78.957	90.208

**Rotated Component Matrix^a^**				
**b**	**Component**		
	**1**	**2**	**3**

VA	−**0.647**	0.571	–0.372
BA	0.054	**0.842**	–0.190
VBR	−**0.809**	–0.161	–0.264
S	–0.053	–0.228	**0.912**
T	0.142	**0.879**	–0.068
DO	–0.061	**0.726**	0.612
NO2	**0.848**	–0.101	–0.274
DOC	**0.894**	0.183	–0.158
Eigenvalue	2.624	2.48	1.528
Variance(%)	32.803	31.003	19.106
Cumulative variance(%)	32.803	63.806	82.912

*Bold Values represent the variables that mainly influence the corresponding principal component.*

## Discussion

### The Subsurface VBR Maximum

**FIGURE 7:**
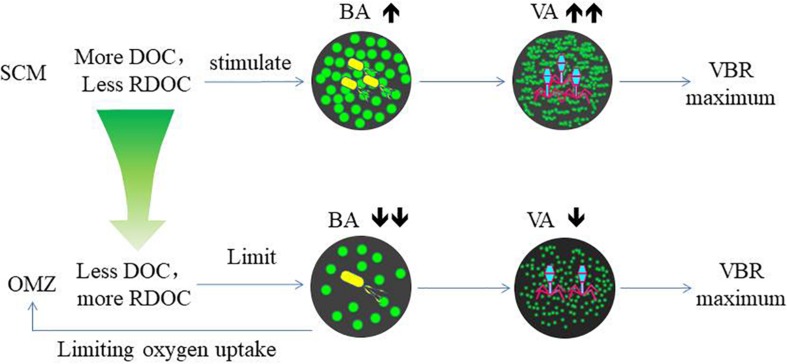
The relationship among DOC, bacteria, and viruses in the SCM and OMZ.

The variability of viral abundance is largely affected by bacteria; however, it is also influenced by other organisms as well as environmental factors. Viral abundance is known to fluctuate with bacterial variation, which is coupled with organic matter supply. The subsurface chlorophyll maximum (SCM, also known as the deep chlorophyll maximum, DCM) exists near the bottom of the surface mixed layer or the top of the pycnocline and nutricline in the water column in oligotrophic oceans (Cullen, 2015); it is also a common permanent feature in the SCS observed in many studies (Lu et al., 2010; Wang et al., 2015). As phytoplankton have access to nutrients at the nutricline, they can carry out photosynthesis and produce organic matter at the SCM layer. The maximum photosynthetic rate is just above SCM versus that in the surface mixed layer (Ghai et al., 2010), and as a result, the extracellular production of organic carbon is also the highest in the photosynthetic activity maximum zone (Avril, 2002). The subsurface DO maximum (Figure 2) above the depth of the chlorophyll maximum in our study indicates the presence of the maximum photosynthetic activity zone. The increased organic matter thus increases bacterial activities and stimulates viral infection and viral abundance in the SCM layer (Santinelli et al., 2010). As a result, this sequence of organic carbon-bacteria-viruses could result in the VBR maximum observed in our study (Figure 7). Our previous study found a significant and positive correlation between the viral abundance and chlorophyll concentration in the northern SCS (He et al., 2009). The more rapid increase in viral abundance versus bacterial abundance likely caused the subsurface VBR maximum. The observation of the subsurface maximum of viral abundance is consistent with other studies. The subsurface maximum has been reported to occur at varying depths from 15 m (Maranger and Bird, 1995) to 150 m (Cochlan et al., 1993; Hara et al., 1996) and is related to discontinuities in the water column (e.g., the pycnocline) or the gradients of chemical and biological parameters (e.g., the nutricline or SCM) (Weinbauer, 2004). Viral phage production and the frequency of bacteria containing mature phages increased with bacterial abundances (Steward et al., 1992; Weinbauer et al., 1993). Vertical profiles showing the maximum viral abundance were also reported in the Southern California Bight (Cochlan et al., 1993), the southeastern Gulf of Mexico (Boehme et al., 1993) and the Northern Adriatic Sea (Weinbauer and Peduzzi, 1995). In our study, the subsurface virus maximum usually occurred above the depth of the SCM at 75 m (E409, E407) to 100 m (E703). Both the depths of the subsurface virus maximum and SCM increased when the water column became deeper and further offshore.

### The Deep VBR Maximum

Herndl and Reinthaler (2013) noted that the function of deep-sea microbial community is fundamentally different from that of surface water communities. One of the concepts to measure the efficiency of bacterial utilization of organic matter is the remineralization length scale in the OMZ. With regard to the abnormally high remineralization length scale, one of the five hypotheses is a low utilization rate of sinking organic matter by microbes, as summarized by Cavan et al. (2017). Our observation suggests that one mechanism is viral control of bacterial activities, which slows down the utilization of sinking organic matter. In open oceans, the oxygen minimum is a result of bacterial consumption of oxygen by bacterial utilization of organic particles, which sink through the subsurface pycnocline and slow down through the deep pycnocline between 700 and 1200 m (Paulmier and Ruiz-Pino, 2009; Liu et al., 2011; Cavan et al., 2017). In the SCS, the OMZ is a permanent feature, as is often observed in other studies (Qu et al., 2006; Wang et al., 2017; Wang et al., 2018). Because the seawater of western Pacific Ocean invades the SCS through the Luzone Strait over the 2400 m sill (Qu et al., 2006; Wang et al., 2018), it brings in the OMZ water mass and moves westward with it (Yang, 1991; Liu et al., 2011). However, the thickness of the OMZ in the western part of the SCS is much thicker than that across the Luzone Strait along the same latitude, and this indicates that local biological activities contribute to the vertical expansion of the OMZ in the SCS. The DO concentrations in the minimum zone varied from 2.72 to 3.21 mg L^–1^ in our study, comparable with other studies which have reported 2.00 ml L^–1^, (1.40 mg L^–1^, Yang, 1991), and 83.50 μmol L^–1^, (2.66 mg L^–1^, Liu et al., 2011). However, DO has rarely been depleted in the OMZ. This suggests that the biological consumption of DO is limited by *in situ* factors, considering that the intermediate water mass has over at least 40 years’ residence time (Li and Qu, 2006).

The coincidence of deep VBR maximum and the OMZ at the same depth suggests their coupling association. Compared with the SCM, the decrease in both bacterial and viral abundance in the OMZ indicates the substrate limitation. The NO_2_ maximum indicates the occurrence of denitrification, suggesting oxygen limitation as reported by other studies (Ganesh et al., 2014). Bacterial growth is limited by refractory DOC, since DOC is estimated to be 3700 and 6000 years old in the North Atlantic and North Pacific Oceans, respectively (Loh et al., 2004). As bacterial phages do not see the substrate limitation, they maintain the same lytic rate. A recent study that synthesizes many data sets from various ecosystems found that the slope between viral (*Y*-axis) versus bacterial abundance (*X*-axis) tilts higher (becoming more horizontal) near lower bacterial abundance, signifying that VBR is relatively higher when bacterial abundance decreases (Knowles et al., 2016). In the study by Arrieta et al. (2015), viruses increased when the concentrated bacterial abundance collapsed due to the substrate limitation, increasing VBR dramatically. These studies support our notion of the substrate limitation of bacterial growth, causing the maximum VBR in the OMZ. In the laboratory study, when the substrate in the culture was limited, viruses lysed bacterial lysates supported more bacterial cell growth (Liu et al., 2015), suggesting more recycling of the substrate. The same study also found the respiration per bacterial cell increased with viruses compared with the control without viruses. This also supports our hypothesis of the viral control of bacterial depletion of DO when organic matter is limited.

The high VBR in the bathypelagic waters reported from the open North Atlantic (Parada et al., 2007; De Corte et al., 2010; De Corte et al., 2012), the South Atlantic Ocean (De Corte et al., 2016), and the Pacific (Yang et al., 2014). The formation of the maximum VBR can also have contributions from responses to other factors influencing bacteria and viruses. A possible explanation of the high VBP at depth is a longer viral turnover time (that is, lower decay rates) in deeper waters than in the surface waters where the viruses remain infective for 1–2 day (Wilhelm et al., 1998; Yang et al., 2014; De Corte et al., 2016). Another possible factor is the physical transport of viruses attached to sinking particles from the euphotic layers and subsequent dissociation in the deeper waters (Proctor and Fuhrman, 1991; Taylor et al., 2003; Bochdansky et al., 2010; Yang et al., 2014). Temperature can affect changes in VBR in the water column; the decay rates of viral assemblages increased between 4 and 25°C, suggesting a positive effect of decreasing temperature on the survival of viruses (Cottrell and Suttle, 1995; Garza and Suttle, 1998; Wei et al., 2018). Therefore, the decreased temperature of 12°C in the mesopelagic waters of the SCS may lead to an increase in the survival rate of viruses at 800–1000 m. Viral abundance and VBR have been reported to be high during anoxia events in deep waters of the Cariaco Trench (Taylor et al., 2001) and Mediterranean Sea (Weinbauer et al., 2003), which supports our notion of the viral control of bacterial activities because anoxia often indicates depletion of organic matter as a result of organic matter consumption. The decreased organic concentrations associated with the DO minimum limited the bacterial abundance. The long viral turnover time, the sinking-particle transport, the lower temperature and near-hypoxic waters favoring viral survival are responsible for the deep VBR maximum observed.

### Biogeochemical Implications of the Findings

The decomposition of host cells by marine virus releases DOC and particulate organic carbon (POC) back into the environment, where they can be absorbed by microorganisms or exported from surface water to the deep ocean (Figure 7). This virus-mediated organic matter recycling process is known as the “viral shunt” (Wilhelm and Suttle, 1999). This in turn affects nutrient cycle and alters the way of OC utilized by prokaryotes (Fuhrman, 1999; Wilhelm and Suttle, 1999; Wommack and Colwell, 2000; Weinbauer, 2004; Suttle, 2005).

Refractory dissolved organic carbon (RDOC) in the deep ocean is the largest carbon pool on the earth (Siegenthaler and Sarmiento, 1993) and very old (Santinelli et al., 2010). In the North Atlantic and North Pacific Oceans, the weighted mean turnover time for DOC in deep-water estimated by δ^14^C, is 3700 and 6000 years, respectively (Loh et al., 2004). However, recently, a new study investigating the dilution hypothesis found that it is the very low DOC concentrations that limit bacterial utilization in the deep ocean (Arrieta et al., 2015). In their incubation experiments, the viral abundance increased after the bacterial abundance declined after the bacterial abundance reached a plateau. This suggests that viral abundance is stimulated by bacterial growth, and the increasing rate of viral abundance exceeded that of bacterial abundance when the substrate became limited again. The finding supports our hypothesis of the viral control of bacterial abundance and bacterial respiration in the OMZ, indicated by the deep maximum VBR. In the OMZ, DOC is very low, such that bacterial growth is more limited than in both the upper and lower zones. However, viruses are less limited by the low DOC and continue to lyse bacteria. This could result in the VBR maximum. Zhang et al. (2014) recently commented on the role of viruses and suggested that viruses can kill the winner “bacteria,” and therefore, leave more DOC in the water column. The VBR maximum in the deep ocean appears to corroborate this notion and suggests that viruses can control bacterial growth due to the already reduced DOC associated with the oxygen minimum and slow down further utilization of DOC, which would otherwise be reduced and consume more oxygen.

## Data Availability

The datasets generated for this study are available on request to the corresponding author.

## Author Contributions

LH collected and measured the samples and drafted the manuscript under the supervision of KY. KY provided the conceptual framework of the study. XY analyzed the DOC data.

## Conflict of Interest Statement

The authors declare that the research was conducted in the absence of any commercial or financial relationships that could be construed as a potential conflict of interest.

## References

[B1] Arrieta J. M. Mayol E. Hansman R. L. Herndl G. J. Dittmar T. Duarte C. M. (2015). Dilution limits dissolved organic carbon utilization in the deep ocean. *Science* 350 1483–1483. 10.1126/science.1258955 26680189

[B2] Avril B. (2002). DOC dynamics in the northwestern Mediterranean Sea (DYFAMED site). *Deep Sea Res. Part II* 49 2163–2182. 10.1016/s0967-0645(02)00033-4

[B3] Bochdansky A. B. van Aken H. M. Herndl G. J. (2010). Role of macroscopic particles in deep-sea oxygen consumption. *Proc. Natl. Acad. Sci. U.S.A.* 107 8287–8291. 10.1073/pnas.0913744107 20406905 PMC2889554

[B4] Boehme J. Frischer M. E. Jang S. C. Kellogg C. A. Pichard S. Rose J. B. (1993). Viruses, bacterioplankton, and phytoplankton in the southeastern Gulf of Mexico: distribution and contribution to oceanic DNA pools. *Mar. Ecol. Prog. Ser.* 97 1–10. 10.3354/meps097001

[B5] Cavan E. L. Trimmer M. Shelley F. Sanders R. (2017). Remineralization of particulate organic carbon in an ocean oxygen minimum zone. *Nat. Commun.* 8:14847. 10.1038/ncomms14847 28322218 PMC5364423

[B6] Cochlan W. P. Wikner J. Steward G. F. Smith D. C. Azam F. (1993). Spatial distribution of viruses, bacteria, and chlorophyll a in neritic, oceanic and estuarine environments. *Mar. Ecol. Prog. Ser.* 92 77–87. 10.3354/meps092077

[B7] Cottrell M. T. Suttle C. A. (1995). Dynamics of a lytic virus infecting the photosynthetic marine picoflagellate Micromonas pusilla. *Limnol. Oceanogr.* 40 730–739. 10.4319/lo.1995.40.4.0730

[B8] Cullen J. J. (2015). Subsurface chlorophyll maximum layers: enduring enigma or mystery solved? *Annu. Rev. Mar. Sci.* 7 207–239. 10.1146/annurev-marine-010213-135111 25251268

[B9] De Corte D. Sintes E. Winter C. Yokokawa T. Reinthaler T. Herndl G. J. (2010). Links between viral and prokaryotic communities throughout the water column in the (sub) tropical Atlantic Ocean. *ISME J.* 4 1431–1442. 10.1038/ismej.2010.65 20485386

[B10] De Corte D. Sintes E. Yokokawa T. Lekunberri I. Herndl G. J. (2016). Large-scale distribution of microbial and viral populations in the South Atlantic Ocean. *Environ. Microbiol. Rep.* 8 305–315. 10.1111/1758-2229.12381 26765966 PMC4959534

[B11] De Corte D. Sintes E. Yokokawa T. Reinthaler T. Herndl G. J. (2012). Links between viruses and prokaryotes throughout the water column along a North Atlantic latitudinal transect. *ISME J.* 6 1566–1577. 10.1038/ismej.2011.214 22258100 PMC3400414

[B12] Fonda Umani S. Malisana E. Focaracci F. Magagnini M. Corinaldesi C. Danovaro R. (2010). Disentangling the effect of viruses and nanoflagellates on prokaryotes in bathypelagic waters of the Mediterranean Sea. *Mar. Ecol. Prog. Ser.* 418 73–85. 10.3354/meps08803

[B13] Fuhrman J. A. (1999). Marine viruses and their biogeochemical and ecological effects. *Nature* 399 541–548. 10.1038/21119 10376593

[B14] Ganesh S. Parris D. J. DeLong E. F. Stewart F. J. (2014). Metagenomic analysis of size-fractionated picoplankton in a marine oxygen minimum zone. *ISME J.* 8 187–211. 10.1038/ismej.2013.144 24030599 PMC3869020

[B15] Garza D. R. Suttle C. A. (1998). The effect of cyanophages on the mortality of Synechococcus sp. and selection for UV resistant viral communities. *Microb. Ecol.* 36 281–292. 10.1007/s002489900115 9852508

[B16] Ghai R. Martin-Cuadrado A. B. Molto A. G. Heredia I. G. Cabrera R. Martin J. (2010). Metagenome of the Mediterranean deep chlorophyll maximum studied by direct and fosmid library 454 pyrosequencing. *ISME J.* 4 1154–1166. 10.1038/ismej.2010.44 20393571

[B17] Hara S. Koike I. Terauchi K. Kamiya H. Tanoue E. (1996). Abundance of viruses in deep oceanic waters. *Mar. Ecol. Prog. Ser.* 145 269–277. 10.3354/meps145269

[B18] He L. Yin K. Yuan X. Li D. Zhang D. Harrison P. J. (2009). Spatial distribution of viruses, bacteria and chlorophyll in the northern south china sea. *Aquat. Microb. Ecol.* 54 153–162. 10.3354/ame01263

[B19] Herndl G. J. Reinthaler T. (2013). Microbial control of the dark end of the biological pump. *Nat. Geosci.* 6 718–724. 10.1038/ngeo1921 24707320 PMC3972885

[B20] Knap A. H. Michaels A. F. Close A. R. Ducklow H. W. Dickson A. G. (1996). *Protocols for the Joint Global Ocean Flux Study (JGOFS) Core Measurements. JGOFS Report no. 19. Reprint of the IOC Manuals and Guides no. 29.* Paris: UNESCO.

[B21] Knowles B. Silveira C. B. Bailey B. A. Barott K. Rohwer F. (2016). Lytic to temperate switching of viral communities. *Nature* 531 466–470. 10.1038/nature17193 26982729

[B22] Lara E. Vaque D. Sa E. L. Boras J. A. Gomes A. Borrull E. (2017). Unveiling the role and life strategies of viruses from the surface to the dark ocean. *Sci. Adv.* 3:e1602565. 10.1126/sciadv.1602565 28913418 PMC5587022

[B23] Li L. Qu T. (2006). Thermohaline circulation in the deep South China Sea basin inferred from oxygen distributions. *J. Geophys. Res. Oceans* 111:C05017 10.1029/2005JC003164

[B24] Liu H. Yuan X. Xu J. Harrison P. J. He L. Yin K. (2015). Effects of viruses on bacterial functions under contrasting nutritional conditions for four species of bacteria isolated from Hong Kong waters. *Sci. Rep.* 5:14217. 10.1038/srep14217 26404394 PMC4585901

[B25] Liu K. K. Chao S. Y. Shaw P. T. Gong G. C. Chen C. C. Tang T. Y. (2002). Monsoon-forced chlorophyll distribution and primary production in the south china sea: observations and a numerical study. *Deep Sea Res. Part I* 49 1387–1412. 10.1016/s0967-0637(02)00035-3

[B26] Liu Y. Bao X. W. Wu D. X. (2011). Analysis of vertical structure and seasonal variation of the dissolved oxygen in the South China Sea. *Period. Ocean U. China* 41 25–32.

[B27] Loh A. N. Bauer J. E. Druffel E. R. M. (2004). Variable ageing and storage of dissolved organic components in the open ocean. *Nature* 430 877–881. 10.1038/nature02780 15318218

[B28] Lu Z. Gan J. Dai M. Cheung A. Y. Y. (2010). The influence of coastal upwelling and a river plume on the subsurface chlorophyll maximum over the shelf of the northeastern South China Sea. *J. Mar. Syst.* 82 35–46. 10.1016/j.jmarsys.2010.03.002

[B29] Magagnini M. Corinaldesi C. Monticelli L. S. De Domenico E. Danovaro R. (2007). Viral abundance and distribution in mesopelagic and bathypelagic waters of the Mediterranean Sea. *Deep Sea Res. Part I* 54 1209–1220. 10.1016/j.dsr.2007.05.006

[B30] Magiopoulos I. Pitta P. (2012). Viruses in a deep oligotrophic sea: seasonal distribution of marine viruses in the epi-, meso- and bathypelagic waters of the eastern mediterranean sea. *Deep Sea Res. Part I* 66 1–10. 10.1016/j.dsr.2012.03.009

[B31] Maranger R. Bird D. F. (1995). Viral abundances in aquatic systems: a comparison between marine and fresh waters. *Mar. Ecol. Prog. Ser.* 121 217–226. 10.3354/meps121217

[B32] Ning X. Li W. K. W. Cai Y. (2005). Standing stock and community structure of photosynthetic picoplankton in the northern South China Sea. *Acta Oceanol. Sin.* 24 57–76.

[B33] Noble R. T. Fuhrman J. A. (1998). Use of SYBR Green I for rapid epifluorescence counts of marine viruses and bacteria. *Aquat. Microb. Ecol.* 14 113–118. 10.3354/ame014113

[B34] Parada V. Sintes E. Aken H. M. V. Weinbauer M. G. Herndl G. J. (2007). Viral abundance, decay, and diversity in the meso- and bathypelagic waters of the North Atlantic. *Appl. Environ. Microbiol.* 73 4429–4438. 10.1128/aem.00029-07 17496133 PMC1932831

[B35] Parsons T. R. Maita Y. Lalli C. M. (1984). *A Manual of Chemical and Biological Methods for Seawater Analysis.* Oxford: Pergamon Press, 173.

[B36] Paulmier A. Ruiz-Pino D. (2009). Oxygen minimum zones (OMZs) in the modern ocean. *Prog. Oceanogr.* 80 113–128. 10.1016/j.pocean.2008.08.001

[B37] Proctor L. M. Fuhrman J. A. (1991). Roles of viral infection in organic particle flux. *Mar. Ecol. Prog. Ser.* 69 133–142. 10.3354/meps069133 15458233

[B38] Qu T. Girton J. B. Whitehead J. A. (2006). Deepwater overflow through Luzon Strait. *J. Geophys. Res. Oceans* 111:C01002. 10.1029/2005JC003139 30254370

[B39] Santinelli C. Nannicini L. Seritti A. (2010). DOC dynamics in the meso and bathypelagic layers of the Mediterranean Sea. *Deep Sea Res. Part II* 57 1446–1459. 10.1016/j.dsr2.2010.02.014

[B40] Shaw P. T. Chao S. Y. (1994). Surface circulation in the South China Sea. *Deep Sea Res. Part I* 41 1663–1683.

[B41] Siegenthaler U. Sarmiento J. I. (1993). Atmospheric carbon dioxide and the ocean. *Nature* 365 119–125.

[B42] Steward G. F. Wikner J. Cochlan W. P. Smith D. C. Azam F. (1992). Estimation of virus production in the sea: II. Field results. *Mar. Microb. Food Webs* 6 79–90.

[B43] Suttle C. A. (2005). Viruses in the sea. *Nature* 437 356–361. 10.1038/nature04160 16163346

[B44] Taylor G. T. Hein C. Iabichella M. (2003). Temporal variations in viral distributions in the anoxic Cariaco Basin. *Aquat. Microb. Ecol.* 30 103–116. 10.3354/ame030103

[B45] Taylor G. T. Iabichella M. Ho T. Y. (2001). Chemoautotrophy in the redox transition zone of the cariaco basin: a significant midwater source of organic carbon production. *Limnol. Oceanogr.* 46 148–163. 10.4319/lo.2001.46.1.0148

[B46] Wang A. Du Y. Peng S. Liu K. Huang R. X. (2018). Deep water characteristics and circulation in the South China Sea. *Deep Sea Res. Part I* 134 55–63. 10.1016/j.dsr.2018.02.003

[B47] Wang N. Huang B. Q. Dong Y. T. Xie X. (2017). The evolution of deepwater dissolved oxygen in the northern South China Sea since 400 ka. *Palaeoworld* 27 301–308. 10.1016/j.palwor.2017.11.001

[B48] Wang S. Li S. Hu J. Geng B. (2015). Experiments in optimizing simulations of the subsurface chlorophyll maximum in the South China Sea. *J. Mar. Syst.* 156 1–15. 10.1016/j.jmarsys.2015.11.003

[B49] Wei W. Zhang R. Peng L. Liang Y. Jiao N. (2018). Effects of temperature and photosynthetically active radiation on virioplankton decay in the western Pacific Ocean. *Sci. Rep.* 8:1525. 10.1038/s41598-018-19678-3 29367730 PMC5784127

[B50] Weinbauer M. G. (2004). Ecology of prokaryotic viruses. *FEMS Microbiol. Rev.* 28 127–181. 10.1016/j.femsre.2003.08.001 15109783

[B51] Weinbauer M. G. Fuks D. Peduzzi P. (1993). Distribution of viruses and dissolved DNA along a coastal trophic gradient in the northern Adriatic Sea. *Appl. Environ. Microb.* 59 4074–4082. 16349109 10.1128/aem.59.12.4074-4082.1993 PMC195869

[B52] Weinbauer M. G. Ingrid B. Manfred G. H. (2003). Lysogeny and virus-induced mortality of bacterioplankton in surface, deep, and anoxic marine waters. *Limnol. Oceanogr.* 48 1457–1465. 10.4319/lo.2003.48.4.1457

[B53] Weinbauer M. G. Peduzzi P. (1995). Significance of viruses versus heterotrophic nanoflagellates for controlling bacterial abundance in the northern Adriatic Sea. *J. Plankton Res.* 17 1851–1856. 10.1093/plankt/17.9.1851

[B54] Wigington C. H. Sonderegger D. Brussaard C. P. Buchan A. Finke J. F. Fuhrman J. A. (2016). Re-examination of the relationship between marine virus and microbial cell abundances. *Nat. Microbiol.* 1:15024. 10.1038/nmicrobiol.2015.24 27572161

[B55] Wilhelm S. W. Suttle C. A. (1999). Viruses and nutrient cycles in the sea. *Bioscience* 49 781–788. 10.2307/1313569

[B56] Wilhelm S. W. Weinbauer M. G. Suttle C. A. Jeffrey W. H. (1998). The role of sunlight in the removal and repair of viruses in the sea. *Limnol. Oceanogr.* 43 586–592. 10.4319/lo.1998.43.4.0586

[B57] Winter C. Kerros M. E. Weinbauer M. G. (2009). Seasonal and depth-related dynamics of prokaryotes and viruses in surface and deep waters of the northwestern Mediterranean Sea. *Deep Sea Res. Part I* 56 1972–1982. 10.1016/j.dsr.2009.07.003

[B58] Wommack K. E. Colwell R. R. (2000). Virioplankton in aquatic ecosystems. *Microbiol. Mol. Biol. Rev.* 64 69–114. 10.1128/mmbr.64.1.69-114.2000 10704475 PMC98987

[B59] Wu J. Chung S. W. Wen L. S. Liu K. K. Chen Y. L. L. Chen H. Y. (2003). Dissolved inorganic phosphorus, dissolved iron, and Trichodesmium in the oligotrophic South China Sea. *Global Biogeochem. Cycles* 17 8–10.

[B60] Yang J. D. (1991). Minimum values of dissolved oxygen vertical distribution in the Central South China Sea. *Oceanol. Limnol. Sin.* 22 353–359.

[B61] Yang Y. Yokokawa T. Motegi C. Nagata T. (2014). Large-scale distribution of viruses in deep waters of the Pacific and Southern Oceans. *Aquat. Microb. Ecol.* 71 193–202. 10.3354/ame01677

[B62] Zhang R. Wei W. Cai L. L. (2014). The fate and biogeochemical cycling of viral elements. *Nat. Rev. Microbiol.* 12 850–851. 10.1038/nrmicro3384 25396723

